# Berberine Inhibits the Adhesion of *Candida albicans* to Vaginal Epithelial Cells

**DOI:** 10.3389/fphar.2022.814883

**Published:** 2022-02-28

**Authors:** Ting Zhao, Kang Zhang, Gaoxiang Shi, Kelong Ma, Benfan Wang, Jing Shao, Tianming Wang, Changzhong Wang

**Affiliations:** ^1^ Department of Pathogenic Biology and Immunology, College of Integrated Chinese and Western Medicine (College of Life Science), Anhui University of Chinese Medicine, Hefei, China; ^2^ Institute of Integrated Traditional Chinese and Western Medicine, Anhui University of Chinese Medicine, Hefei, China; ^3^ Anhui Province Key Laboratory of Chinese Medicinal Formula, Hefei, China

**Keywords:** Berberine, *Candida albicans*, vaginal epithelial cells, vulvovaginal candidiasis, adhesion

## Abstract

Vulvovaginal candidiasis (VVC) is an inflammatory disease of the vagina mainly caused by *Candida albicans* (*C. albicans*), which affects around three-quarters of all women during their reproductive age. Although some antifungal drugs such as azoles have been applied clinically for many years, their therapeutic value is very limited due to the emergence of drug-resistant strains. Previous studies have shown that the adhesion of *C. albicans* to vaginal epithelial cells is essential for the pathogenesis of VVC. Therefore, preventing the adhesion of *C. albicans* to vaginal epithelial cells may be one of the most effective strategies for the treatment of VVC. Berberine (BBR) is a biologically active herbal alkaloid that was used to treat VVC. However, so far, its mechanism has remained unclear. This study shows BBR significantly inhibits the adhesion of *C. albicans* to vaginal epithelial cells by reducing the expressions of ICAM-1, mucin1, and mucin4 in vaginal epithelial cells, which play the most important role in modulating the adhesion of *C. albicans* to host cells, and balancing IL-2 and IL-4 expressions, which play a key effect on regulating the inflammatory response caused by *C. albicans* infection. Hence, our findings demonstrate that BBR may be a potential therapeutic agent for VVC by interfering with the adhesion of *C. albicans* to vaginal epithelial cells and represents a new pathway for developing antifungal therapies agents from natural herbs.

## Introduction

*Candida albicans*, a yeast-hypha dimorphic fungus, is widely found in the vagina, gastrointestinal tract, oral cavity, and on skin surface (Richardson et al., 2019), which easily results in opportunistic infection under certain circumstances and causes diseases such as Vulvovaginal Candidiasis (VVC). Adhesion of *C. albicans* to host cells is a key link for the opportunistic infection caused by this fungus (Mayer et al., 2013; Wu et al., 2020), which makes it prone to survive in the vagina, oral cavity, or other body parts (Krachler and Orth 2013). Multiple mechanisms are involved in the regulation of this adhesion. Studies have shown that *C. albicans* infection can stimulate vaginal epithelial cells to highly express adhesion molecules, such as ICAM-1, mucin1, and mucin4 (Moncla et al., 2016; Mikamo et al., 2018). Apparently, these increasingly regulatory molecules can create a favorable microenvironment for the colonization of *C. albicans* and the following disorders of local immune homeostasis. Therefore, reducing these adhesion molecules may block adhesion of *C. albicans* to host cells and protect the host from diseases caused by the opportunistic fungal infection.

VVC is an infectious disease of the female reproductive tract mainly caused by *C. albicans*, which can affect up to 75% of women of child-bearing age. The occurrence of VVC is related to the crucial virulence factors of *C. albicans,* such as secreted aspartyl proteinases (Sap), lipases (Lip), phospholipases (PL), candidalysin, morphological transition, and biofilm formation (Richardson et al., 2018; Willems et al., 2020). On the other hand, the virulence of *C. albicans* also depends on host immune status, especially the immune defense function of vaginal epithelial cells (Luan et al., 2020). *C. albicans* interacts with epithelial cells through adhesins on the cell wall (Martin et al., 2018). Epithelial cells provide the surface area that enables the initial adhesion of *C. albicans*. Under physiological conditions, this pathogen-host interaction, over long-term evolution, allows *C. albicans* and the host cells to adapt to each other without causing any disease. However, when the damaging effect of *C. albicans* exceeds the protective effect of the vaginal mucosal barrier, the impaired balance between *C. albicans* and the host may lead to infection. The adhesion of *C. albicans* to vaginal mucosal epithelial cells is known to be the initial step for the onset of VVC. Therefore, the inhibition of *C. albicans* adhesion and the protection of vaginal mucosal epithelial cells may be a potential strategy that prevents the VVC occurrence and development. Conventional antifungal drugs, such as fluconazole and echinocandin, mainly target the cell wall or cell membrane, so as to inhibit or even kill fungi, rather than to block fungal adhesion to host cells (Eschenauer, et al., 2015). Some reported that *C. albicans* biofilms could be inhibited by these drugs *in vitro*, but not in the setting of VVC model (Maiolo et al., 2014; Pesee et al., 2016). Given adhesion as the initial stage of biofilm formation, the drugs mentioned above might also interfere with adhesion to a certain extent, but there is still a lack of sufficient evidence in this regard.

Although some antifungal drugs such as azoles have been applied clinically for many years, the inherent resistance of *C. albicans* to these agents limited their therapeutic values (Sobel and Sobel 2018). In addition, these drugs also have side effects, such as liver toxicity. Hence, searching for new antifungal compounds with relatively low toxicity from natural herbs is an alternative pathway to develop new antifungal agents or therapies.

Berberine (BBR), a quaternary ammonium compound, is the most abundant bioactive component found in traditional Chinese herbs *Coptis chinensis Franch [*Ranunculaceae*]* and Phellodendron chinense C.K.Schneid. [Rutaceae]. It has been applied clinically to treat bacterial diarrhea, diabetes, and other diseases. Studies have shown that BBR can function as an antibacterial, antifungal, anti-inflammatory, immunomodulatory, and even anti-cancer agent (Zhang et al., 2010; Li et al., 2014; Pirillo and Catapano 2015; Wang et al., 2018). Among them, the antifungal mechanism of BBR may involve the down-regulation of some key genes related to the integrity of *C. albicans* cell wall after treatment with this compound, such as the hypha-specific gene ECE1 related to the morphological transformation of *C. albicans* (Huang et al., 2020) and the genes FKS1 and FKS2 related to the synthesis of *β*-glucan components of *C. albicans* (Yang et al., 2021). Although BBR is reported to have inhibitory effects on *C. albicans*, to some extent, the detailed mechanism remain unknown (Xie et al., 2020).

In this study, whether BBR is able to suppress VVC pathogenesis by interfering with the interaction between *C. albicans* and host vaginal epithelial cells is investigated. Our results show that BBR significantly inhibits the adhesion of *C. albicans* to vaginal epithelial cells by decreasing the expression of ICAM-1, mucin1, and mucin4 in cells and balancing IL-2 and IL-4 expression during inflammation and shows its potential therapeutic value on VVC. The characterization of the herbal compound involved in the regulation of adhesion in VVC pathogenesis may lead to novel anti-fungal therapies that could significantly improve treatment for VVC reducing the public health burden of such diseases.

## Materials and Methods

### Yeast Strain Culture

*Candida albicans* SC5314 was kindly provided by Professor Jiang Yuanying from the College of Pharmacy, Second Military Medical University (Shanghai, China). The glycerol-preserved fungal strain taken out from the −80°C refrigerator was initially streaked onto Sabouraud’s agar. By using a single colony, the strain was routinely inoculated for three generations in Sabouraud’s agar plates. The strain was then activated and propagated in liquid Sabouraud medium (HopeBio-Tech Co., Qingdao, China) at 37°C for 12–16 h until exponential growth phase was achieved. Revived *C. albicans* cells were pooled by centrifugation at 3,000 ×g. After two washing steps using sterile PBS (Punuosai Life-Tech Co., Wuhan, China), cells were resuspended in RPMI-1640 medium (LifeTechnologies Corporation, United States) and adjusted to a defined cell density using a hemocytometer prior to subsequent tests.

### Cell Culture

A431 human vaginal epidermoid carcinoma cell line (Shanghai Cell Bank of the Chinese Academy of Sciences) was cultured at 37°C with 5% CO_2_ in Dulbecco’s Modified Eagle’s Medium (Hyclone, United States) supplemented with 10% fetal bovine serum (Gibco, United States) and 1% penicillin-streptomycin (Biyuntian Biotechnology Co., Ltd., Shanghai, China). At 24 h prior to experimentation, the culture medium was replaced with serum-free medium and maintained until cell harvest.

### Antifungal Assay

The minimum inhibitory concentration (MIC) of BBR (Chengdu Munster Biotech Co., Ltd., China; purity: 98.38% by HPLC) against *C. albicans* SC5314 was evaluated in 96-well microplates (Corning, United States) using the broth microdilution method described by the Clinical and Laboratory Standard Institute (CLSI) M27-A3 document (CLSI, 2008) (Pierce et al., 2008). Initial yeast inoculum was adjusted to1-5× 10^3^ CFU/ml in PRMI-1640 medium buffered to pH 7.0 with MOPS. The inoculum size was confirmed by plating serially diluted yeast-containing dilutions on Sabouraud agar plates. Each well that contains 100 μl of yeast inoculum and a similar volume of two-fold diluted BBR (16–1,024 μg/ml) was incubated at 37°C for 48 h. In addition, a drug-free control with fungal cells was included. Fluconazole was included as quality control. MIC of BBR was defined as the lowest drug concentration that caused no visible cell growth.

### XTT Reduction Assay

XTT reduction assay was carried out to assess *C. albicans* viability. Briefly, in a 96-well plate, 100 μl of *C. albicans* (2 × 10^6^ CFU/ml) and 100 μl of BBR (32–256 μg/ml) were added into each well and incubated at 37°C for 1, 2 and 3 h. Following two washing steps using sterile PBS, the OD value was detected by XTT reduction assay at 492 nm in a microplate reader (Manoharan et al., 2017; Rossoni et al., 2019).

### Cytotoxicity Test

In order to detect the toxicity of BBR to A431 cells, we treated A431 cells (2 × 10^5^ cells/ml) with BBR at different concentrations. Cells were inoculated in 96-well plates and cultured at 37°C for 24 h before treatment with the same volume of BBR (8–128 μg/ml). Finally, 10 μl of CCK8 solution (Biyuntian Biotechnology Co., Ltd., Shanghai, China) was added into each well and incubated for 1 h, and the absorbance of each well was measured at 450 nm using a microplate reader.

### Adhesion Test

Gram staining, an established staining method for bacteria, was used to observe the adherence of *C. albicans.* Briefly, in a 6-well plate, 2 ml of cells (2 × 10^5^ cells/ml) were cultured for 24 h at 37°C and 5% CO_2_ before incubation with *C. albicans* diluted by the same volume of DMEM culture medium to different concentrations (2 × 10^4^, 2 × 10^5^, 2 × 10^6^, 2 × 10^7^, 2 × 10^8^ CFU/ml, respectively) for 1, 2 and 3 h. At designated time points, cells were fixed with 4% paraformaldehyde at room temperature for 15 min, and washed 3 times with PBS. After that, enhanced Gram staining solution (Beijing Regen Biotechnology Co., Ltd., China) was applied. Finally, oil immersion lens (OLYMPUS BX51, Tokyo) was used for cell imaging.

### Cell Grouping and Berberine Intervention

Gram staining was performed to evaluate the adherence of *C. albicans* with or without BBR treatment. Briefly, in a 6-well plate, 2 ml of A431 cells (2 × 10^5^ cells/ml) were cultured for 24 h at 37°C and 5% CO_2_, then the culture supernatant was removed. Cells receiving different treatments were grouped as follows: Blank group (A431 cells added in DMEM culture medium), Control group (A431 cells added in DMEM culture medium plus *C. albicans*), Treatment group (A431 cells added in DMEM culture medium plus *C. albicans* and BBR), Negative control group (A431 cells added in DMEM medium containing BBR without *C. albicans*). Cells were cultured at 37°C with 5% CO_2_ for 1, 2 and 3 h. At corresponding time points, cells were fixed with 4% paraformaldehyde, followed by washing with PBS and staining with enhanced Gram staining solution. Finally, oil immersion lens (OLYMPUS BX51, Tokyo, Japan) was used for cell imaging.

### Scanning Electron Microscopy

To further detect the adhesion of *C. albicans* to vaginal epithelial cells, samples of fungi-cells adhesion were prepared by using round cover slides (Bioshrp, China) in a 12-well plate for visualization using SEM. Cell grouping and BBR intervention were performed as described above. Samples were fixed by 2.5% glutaraldehyde overnight, and dehydrated by 30, 50, 70, 90, and 100% ethanol for 10 min. Samples were air-dried before sputter coating with gold in a vacuum evaporator. Morphological observation and image acquisition were performed using SEM at ×2000 magnification (SU8100, HITACHI, Japan).

### Immunofluorescence

Calcofluor white (CFW) (Sigma, United States) was used to determine the adhesion of *C. albicans* to vaginal epithelial cells. Samples were prepared in 6-well plates as described above. Briefly, A431 cells were cultured for 1, 2, and 3 h after BBR treatment. At corresponding time points, cells were washed with sterile PBS before staining with 5% CFW (1 mg/ml) at room temperature for 10 min. Cells were observed using DMI8 fluorescence microscope at ×200 magnification (Leica, GER).

To further determine the possible role of intercellular adhesion molecule ICAM-1 in *C. albicans* adhesion to vaginal epithelial cells, cellular localization of expressed ICAM-1 was analyzed. Briefly, cell sample preparation was performed as described above, following which coverslips were taken out and washed 3 times with PBS. Cells were then fixed with 4% paraformaldehyde for 15 min and permeabilized with 0.5% Triton X-100 (Beijing Soleibao Company, China) for 30 min before blocking with 1% BSA (Beijing Soleibao Company, China) for 1 h. After 3 washing steps with PBS, cells were incubated with ICAM-1 primary antibody (1:200 diluted in 1% BSA) overnight at 4°C, and then with FITC-conjugated Goat Anti-Rabbit IgG (Haul) secondary antibody (1:150 diluted in 1% BSA) in the dark at room temperature for 1 h. Finally, 50 μl of anti-fluorescence attenuation sealing agent (including DAPI) (Beijing Soleibao Company, China) was added and incubated at room temperature in the dark for 20 min for cell imaging and quantification using a DMI8 inverted fluorescence microscope (Leica, GER).

### Western Blot

Western blotting was performed to detect the expression of ICAM-1, mucin1 and mucin4. At corresponding time points, culture medium was removed and cells were washed 6 times with PBS before harvest using a cell scraper. After treatment with protein extract solution containing protease inhibitor, total cell protein extract solution was centrifuged at 12,000 rpm, 4°C for 5 min. The protein concentration of cells in each treatment group was determined using BCA Protein Assay Kit. Proteins were separated by SDS-PAGE gel electrophoresis before being transferred onto PVDF membranes (Milipore, United States). Transferred membranes were blocked for 2 h in 5% non-fat dry milk in TBST before incubation overnight at 4°C with one of the following primary antibodies respectively: rabbit anti-β-actin (1:5,000; Abbkine, United States), rabbit anti-ICAM-1 antibody (1:1,000; Zhengneng Biotech Co., Chengdu, China), rabbit anti-Mucin-1 antibody (1:900; Boaosen Biotech Co., Beijing, China) and rabbit anti-Mucin-4 antibody (1:1,000; Zhengneng Biotech Co., Chengdu, China). After three 10-min washing steps with Tween20/TBS, the membranes were incubated with secondary antibody (1:10,000; Affinity, United States) at room temperature for 2 h. After washing three times with Tween20/TBS, the membranes were treated with ECL reagent (Advansta, United States) and visualized using FluorChem M Imaging System (ProteinSimple, United States). Data were quantified by automated densitometry using ImageJ. Densitometric data were normalized against *β*-actin in triplicates.

### ELISA

The levels of cytokines (IL-2 and IL-4) from vaginal epithelial cells were measured by enzyme-linked immunosorbent assay kit. Cell grouping and BBR treatment were performed as described above. At corresponding time points, cell culture supernatant was collected and the levels of IL-2 and IL-4 were analyzed by ELISA (ElabScience, Wuhan, China) according to manufacturer’s instructions.

### Statistical Analysis

Differences between experimental groups were assessed for significance using a two-tailed unpaired Student’s *t*-test and Two-way ANOVA analysis with GraphPad Prism 5 software. All experiments were repeated at least three times. Data are expressed as mean ± standard deviation and *p* < 0.05 is considered to be statistically significant.

## Results

### Determination of the Minimum Inhibitory Concentration of Berberine on *C. albicans*

**FIGURE 1:**
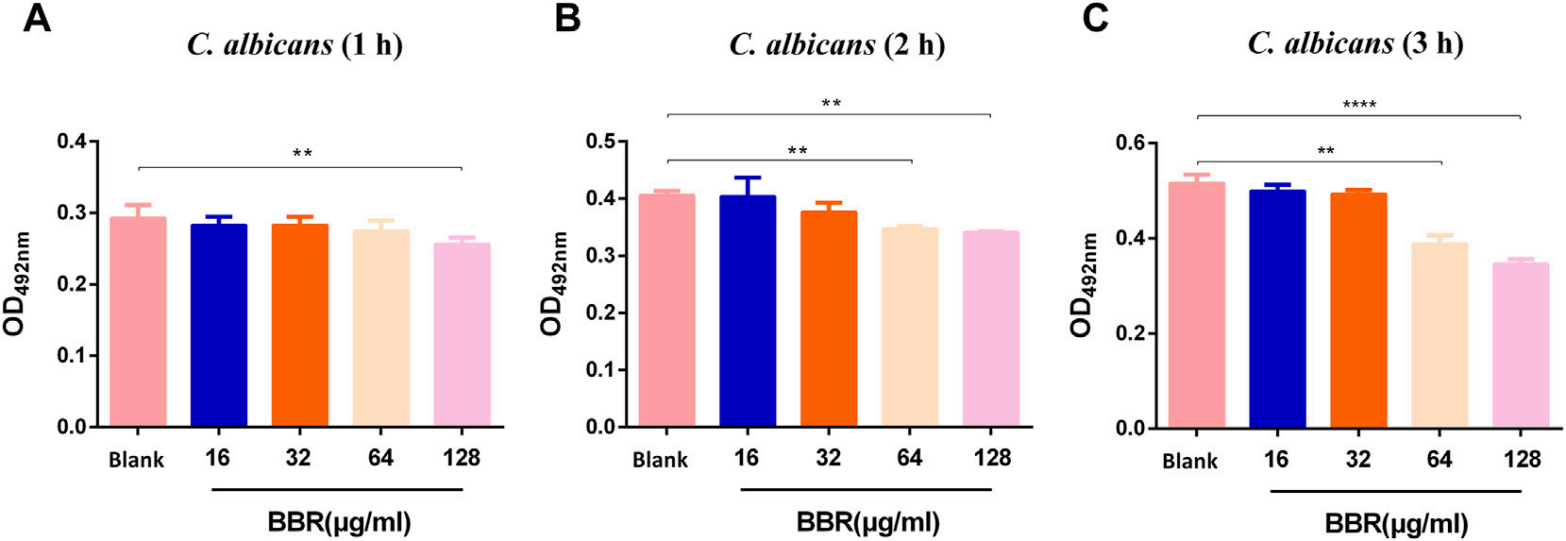
Effect of different concentrations of BBR on the viability of *C. albicans* at **(A)** 1 h **(B)** 2 h, and **(C)** 3 h. The drug-free strain-containing medium was set as the Blank. The drug concentration of treatments was as follows: Blank, 16 µg/ml BBR, 32 µg/ml BBR, 64 µg/ml BBR and 128 µg/ml BBR. Data are shown as mean ± SD. **p < 0.01, ****p < 0.0001 vs. compared with Blank.

To determine the inhibitory effect of BBR on *C. albicans*, the MIC of BBR against *C. albicans* SC5314 was examined. The MIC of BBR for this strain was 64 µg/ml according to the result of two-fold dilution assay, and the MIC of fluconazole against *C. albicans* is 1 µg/ml. To further investigate the effect of BBR on the viability of this strain, an XTT reduction assay was performed. After this strain was treated with different concentrations of BBR ranging from 32 µg/ml to 256 µg/ml for 1, 2, 3 h, the OD value was measured at a wavelength of 492 nm. Our results showed that this compound had no effects on the viability of *C. albicans* at the sub-inhibitory concentrations of 16 and 32 µg/ml (Figures 1A,B), while it could significantly reduce this fungal viability in a dose and time-dependent manner at concentrations higher than 32 µg/ml (Figure 1C). Taken together, our findings indicate that although it shows a certain inhibitory effect at high concentrations, the inhibitory effect of BBR on the growth of *C. albicans* can be neglected when its concentration is lower than 32 µg/ml.

### Determination of Toxicity of Berberine to Vaginal Epithelial Cells

**FIGURE 2:**
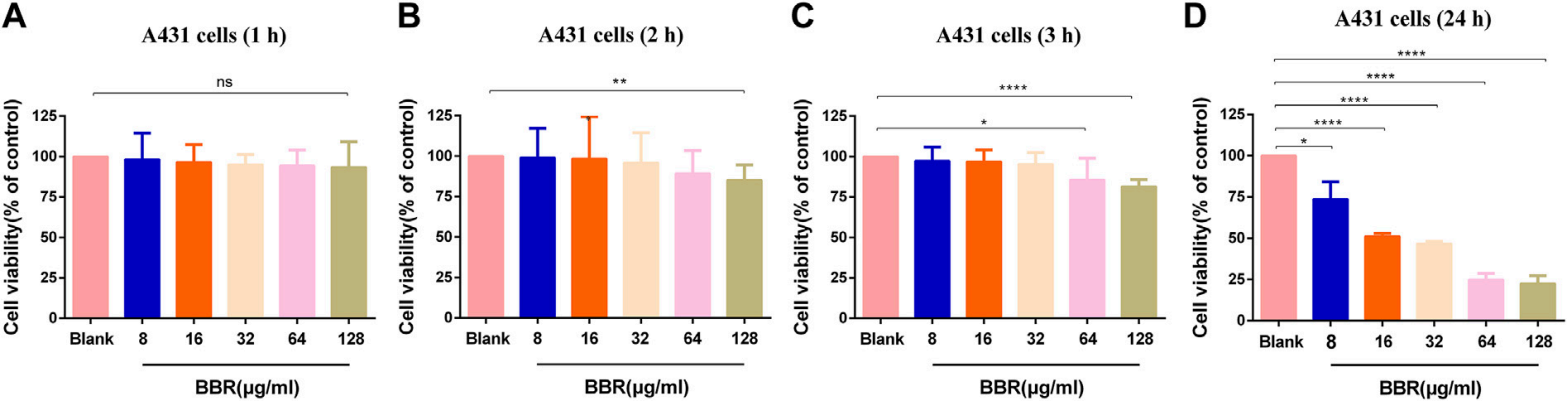
Effect of different concentrations of BBR on the viability of A431 cells at **(A)** 1 h **(B)** 2 h **(C)** 3 h, and **(D)** 24 h. The drug-free cell-containing medium was set as the Blank. The drug concentration of treatments was as follows: Blank, 8 µg/ml BBR, 16 µg/ml BBR, 32 µg/ml BBR, 64 µg/ml BBR and 128 µg/ml BBR. Data are shown as mean ± SD. *p < 0.05, **p < 0.01, ****p < 0.0001. vs. compared with Blank.

**FIGURE 3:**
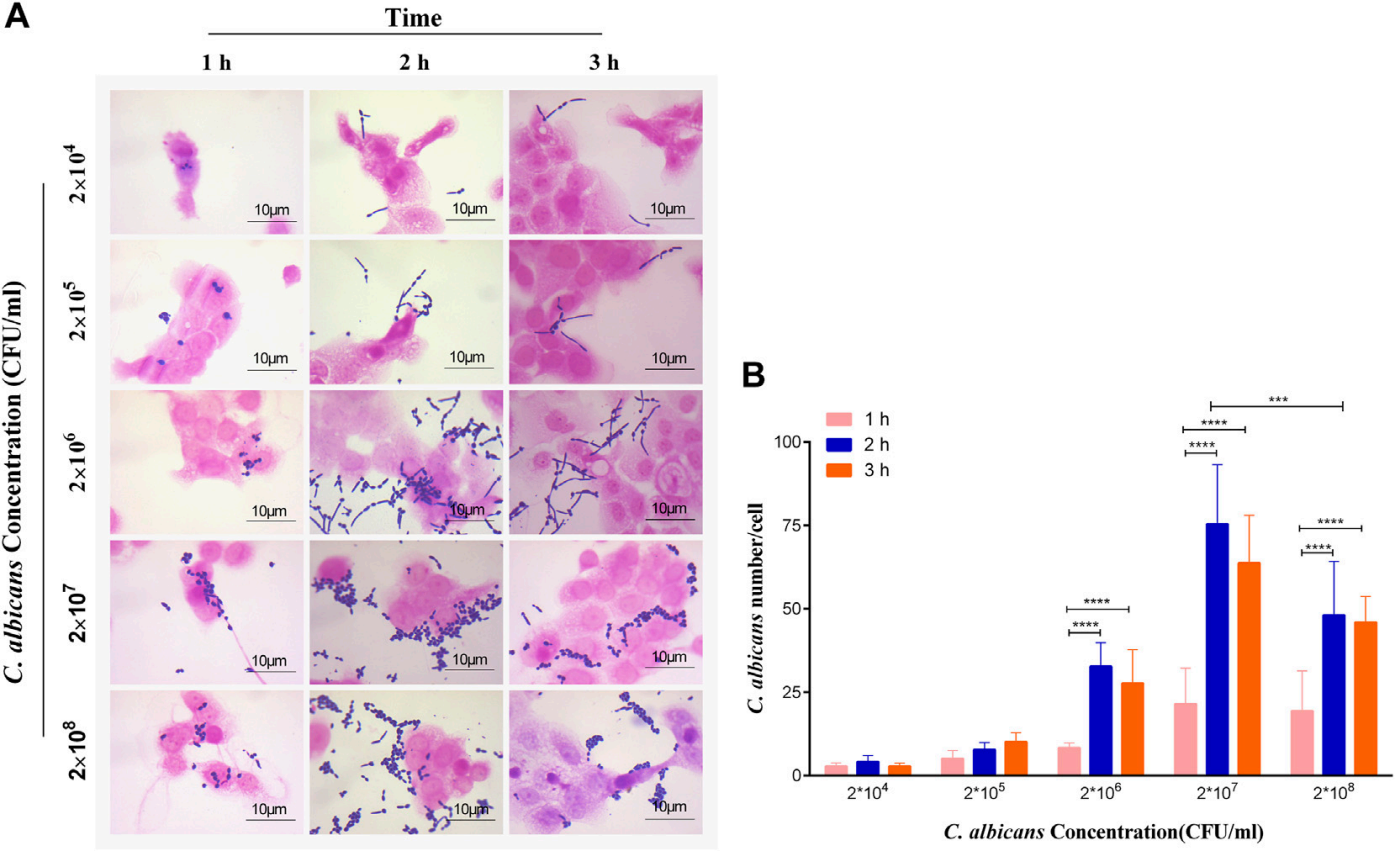
**(A)** Observation and **(B)** quantification of different concentrations of *C. albicans* on its adherence to A431 cells at 1, 2 and 3 h, respectively. ****p < 0.0001. vs. 1 h group. Scale bar, 10 μm.

To determine the toxicity of this compound to vaginal epithelial cells, the viability of these cells after being treated with BBR at different doses and times was measured. The untreated cell group with a cell survival rate of 100% was used as a control for relative comparison. As shown in Figure 2A, there was no change in cell viability after treatment with 8–128 μg/ml BBR for 1 h. However, when they were exposed to 128 μg/ml BBR for 2 h (Figure 2B) or 3 h (Figure 2C) or 64 μg/ml for 3 h (Figure 2C), the viability of these cells was significantly reduced. Furthermore, when the cells were exposed to BBR for 24 h, each dose group could show cytotoxicity (Figure 2D). The above results indicate that the cell survival rate is related to the time and concentration of the compound BBR to treat the cells. For this reason, 32 μg/ml was determined as the appropriate concentration of BBR in the following adhesion experiment.

### Optimal Dose of *C. albicans* for Adhesion to Vaginal Epithelial Cells

To explore the appropriate condition of the adhesion of *C. albicans* to vaginal epithelial cells, the concentration of *C. albicans* and the exposure time of A431 cells to *C. albicans* were considered as two crucial factors. The results were measured by the Gram staining. As shown in Figure 3A, the adhered number of yeasts to A431 cells increased with the increase of *C. albicans* concentrations from 2×10^4^ to 2 × 10^7^ CFU/ml, while the adhesion capability significantly reduced when the concentration of this fungi increased to 2 × 10^8^ CFU/ml. On the other hand, there were no significant differences for the adhesion capability of *C. albicans* to vaginal epithelial cells at different exposure times (1, 2 and 3 h) when the concentration of *C. albicans* was 2 × 10^4^ to 2 × 10^5^ CFU/ml(Figure 3B). However, the adhesion capability significantly increased with the increase of exposure time when the concentration of *C. albicans* increased to 2 × 10^7^ CFU/ml, in which the adhesion number of *C. albicans* to cells peaked with a mean of 75 *C. albicans* per cell (Figure 3B). Therefore, 2 × 10^7^ CFU/ml was considered to be the adhesion concentration of *C. albicans*.

### Effect of Berberine on the Adhesion of *C. albicans* to Vaginal Epithelial Cells

**FIGURE 4:**
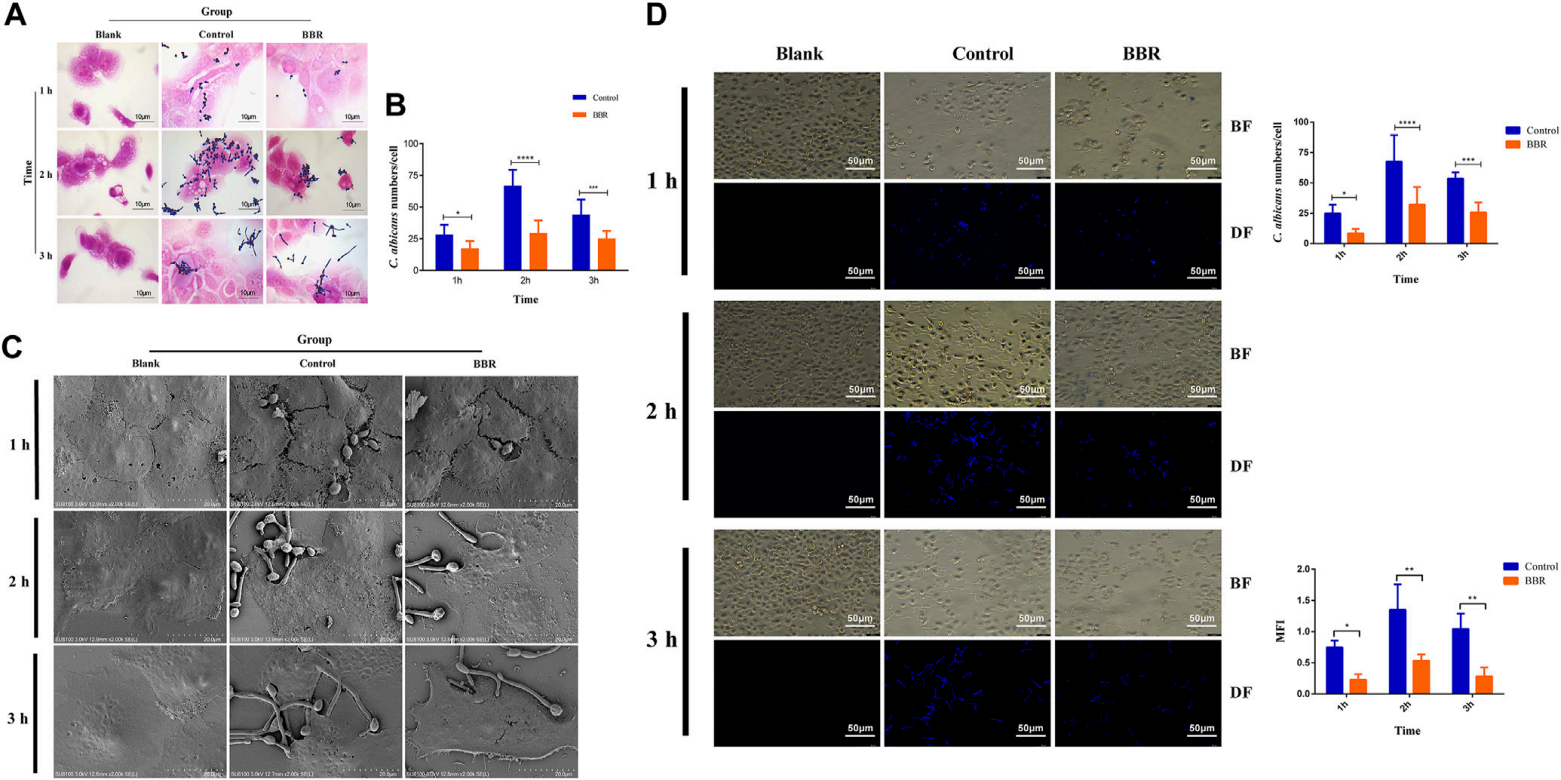
**(A)** Light microscopy image, and **(B)** quantification **(C)** SEM images and **(D)** Fluorescent images, quantification of *C. albicans* numbers adhered to A431 cells and quantification of mean fluorescence intensity of *C. albicans* numbers adhered to A431cells in the Blank, Control, and BBR(32 μg/ml) groups at 1, 2, 3 h, respectively. BF: Bright field, DF: Dark field; *p < 0.05, **p < 0.01, ***p < 0.001, ****p < 0.0001. vs. Control group. Scale bar, 10 μm **(A)**, 20 μm **(C)** and 50 μm **(D)**.

To determine the inhibitory effect of BBR on the adhesion of *C. albicans* to vaginal epithelial cells, 32 μg/ml BBR and 2 × 10^7^ CFU/ml *C. albicans* were added into 2 × 10^5^ A431cells in a 6-well plate. After treatment with different exposure time, the plate was stained by the Gram staining assay. As shown in Figure 4A, compared with the control, the adhesion number of *C. albicans* treated with BBR significantly reduced. It is worth noting that the inhibitory effect of BBR on adhesion of *C. albicans* to A431 cells was time-dependent. Compared with 1 h, the inhibitory effect on adhesion of *C. albicans* to cells was much stronger after treatment with BBR for 2–3 h (Figure 4B). To further observe the inhibitory effect of BBR, the adhesion of *C. albicans* after BBR treatment was visualized by SEM. A similar result with the Gram staining assay was obtained (Figure 4C).

Furthermore, CFW staining was used to evaluate the inhibitory effect of BBR on adhesion of *C. albicans* to A431 cells. The adhesion number of *C. albicans* was counted and the mean fluorescence intensity (MFI) was determined under an inverted fluorescence microscope. As shown in Figure 4D, compared with the control, the adhesion number (right-up panel) and MFI (right-low panel) of *C. albicans* to A431 cells in the group treated with BBR significantly decreased. Also, a stronger inhibitory effect was observed after treatment with BBR for 2 or 3 h, which is similar to the result of the Gram staining assay. Taken together, our data revealed that BBR may play an important role in regulation of adhesion of *C. albicans* to vaginal epithelial cells.

### The Expression of ICAM-1, mucin1, and mucin4 in Vaginal Epithelial Cells Exposed to *C. albicans* Reduced After Treatment With Berberine

**FIGURE 5:**
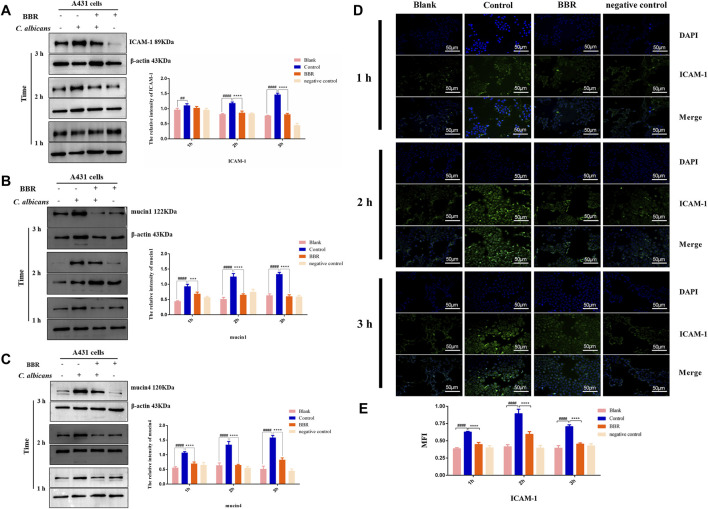
Western blot and quantification of the expression levels of **(A)** ICAM-1 **(B)** mucin1 and **(C)** mucin4 in A431 cells of the Blank, Control, BBR(32 μg/ml) and negative control groups at 1, 2, 3 h, respectively. **(D)** Observation and (B) quantification of BBR on the expression of adhesion molecule ICAM-1 in A431 cells stimulated by *C. albicans* at 1, 2, 3 h, respectively. ##*p* < 0.01, ####*p* < 0.0001. vs. Blank group; ***p < 0.001, ****p < 0.0001. vs. Control group. Scale bar, 50 μm.

The adhesion of *C. albicans* to vaginal epithelial cells involves a complex interaction between the fungi and the cells, which depends not only on the ligands on the surface of *C. albicans*, but also on receptors or adhesion molecules on the surface of host cells. ICAM-1, a cell adhesion molecule, is known to mediate the adhesion of *C. albicans* to vaginal epithelial cells (Youn et al., 2008). To explore the detailed mechanisms that BBR regulates the adhesion of *C. albicans* to vaginal epithelial cells, the expression of ICAM-1 in the A431 cells exposed to *C. albicans* was detected after treatment with BBR. As shown in Figure 5A, compared with A431 cells without any treatment, *C. albicans* could strongly induce the expression of ICAM-1 in A431 cells in a time-dependent manner of *C. albicans* exposure, while its expression significantly reduced after treatment with BBR (*p* < 0.01), which indicates BBR may regulate the adhesion of *C. albicans* to vaginal epithelial cells by blocking ICAM-1. To further verify this regulation, the A431 cells exposed to *C. albicans* were performed an immunofluorescence (IF) analysis after treatment with or without BBR. A similar result was obtained. The IF analysis visualized that the expression of ICAM-1 in the A431 cells exposed to *C. albicans* was significantly higher than that in the A431 cells without *C. albicans* infection with the increase of *C. albicans* adhesion time, while its expression was significantly decreased after treatment with BBR (Figures 5D,E; *p* < 0.0001).

Mucin1 and Mucin4 are another two important molecules that mediate the adhesion of pathogens to vaginal epithelial cells (Moncla et al., 2016). To further verify our finding that BBR can block adhesion of *C. albicans* to vaginal epithelial cells, the expressions of mucin1 and mucin4 were detected by Western blot. Our results showed that the expression levels of mucin1 and mucin4 in the A431 cells exposed to *C. albicans* significantly increased in a time-dependent manner of *C. albicans* exposure (Figures 5B,C; *p* < 0.01). After treatment with BBR, however, the expression of mucin1 and mucin4 in these cells exposed to *C. albicans* significantly decreased, indicating that BBR may block adhesion of *C. albicans* by regulating the expression of mucin1 and mucin4.

Taken together, our findings indicate BBR may block the adhesion of *C. albicans* to vaginal epithelial cells by modulating ICAM-1, mucin1, and mucin4.

### The Balance of IL-2 and IL-4 Was Rebuilt After Treatment With Berberine in Vaginal Epithelial Cells Exposed to *C. albicans*

**FIGURE 6:**
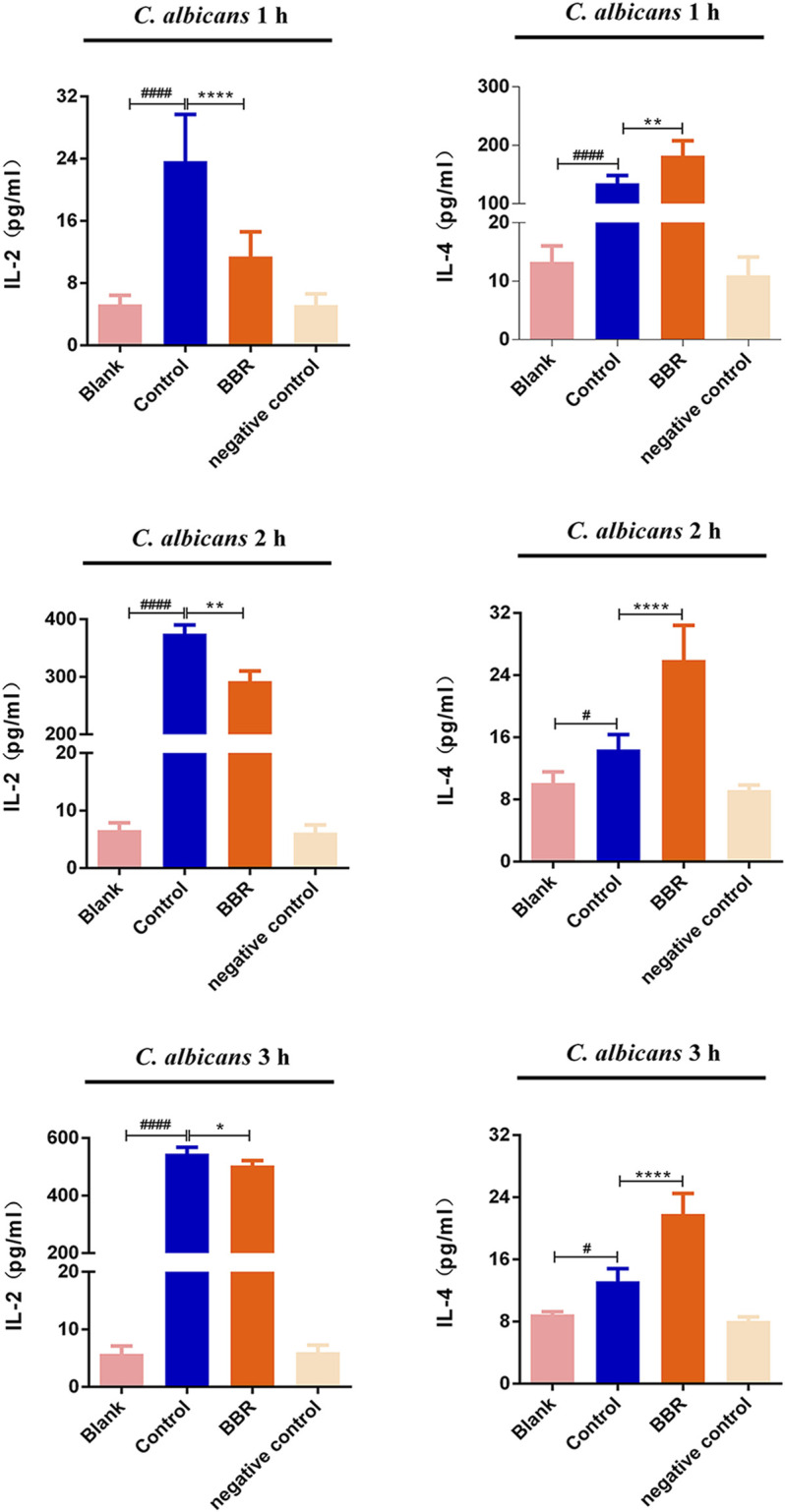
The levels of IL-2 and IL-4 in the Blank, Control, BBR(32 μg/ml) and negative control groups at 1 h (upper), 2 h (middle)and 3 h (lower), respectively. #*p* < 0.05, ####*p* < 0.0001. vs. Blank group; *p < 0.05, **p < 0.01, ****p < 0.0001. vs. Control group. Scale bar, 20 μm.

When VVC occurs, *C. albicans* often induces vaginal epithelial cells to produce a large number of cytokines, including pro-inflammatory factors and anti-inflammatory factors, in which IL-2 and IL-4 are considered as the representative of pro-inflammatory and anti-inflammatory members, respectively. During VVC pathogenesis, the local immune homeostasis of the vagina is often broken. To explore whether the effect of the compound BBR on the local immune homeostasis of the vagina, the levels of IL-2 and IL-4 in A431 cells exposed to *C. albicans* were measured by ELISA assay after treatment with BBR. Our results showed that the levels of IL-2 and IL-4 in A431 cells exposed to *C. albicans* were significantly higher compared with those from A431 cells without *C. albicans* exposure (*p* < 0.05), indicating the local homeostasis of vagina can be broken by *C. albicans* exposure. In the presence of BBR, however, the level of IL-2 significantly decreased and the level of IL-4 was significantly increased (Figure 6; *p* < 0.01). It is worth noting that exposure time of A431 cells to *C. albicans* could affect the expression of IL-2 and IL-4. With extension of exposure time to *C. albicans*, the level of IL-2 secreted by A431 cells significantly increased, while the level of IL-4 significantly decreased (Table 1).

**TABLE 1:** IL-2/IL-4 ratios.

Time	Cytokine (pg/ml)	Blank	Control	BBR	Negative control	*F*-value	*p*-value
—	IL-2	5.28 ± 1.16	23.70 ± 5.98	11.42 ± 3.17	5.19 ± 1.44	24.667	<0.0001^a^
1 h	IL-4	13.27 ± 2.75	134.77 ± 13.20	182.15 ± 25.49	10.98 ± 3.11	142.872	<0.0001^a^
—	IL-2/IL-4	0.40 ± 0.03	0.18 ± 0.06	0.06 ± 0.03	0.51 ± 0.24	10.747	<0.0001
—	IL-2	6.61 ± 1.26	374.81 ± 15.44	292.50 ± 17.69	6.17 ± 1.35	1,062.304	<0.0001^a^
2 h	IL-4	10.12 ± 1.44	14.43 ± 11.94	25.96 ± 4.44	9.21 ± 0.64	36.494	<0.0001^a^
—	IL-2/IL-4	0.65 ± 0.05	26.28 ± 3.10	11.45 ± 1.61	0.67 ± 0.15	192.569	<0.0001^a^
—	IL-2	5.75 ± 1.36	545.62 ± 22.22	504.24 ± 17.79	6.04 ± 1.24	1771.245	<0.0001^a^
3 h	IL-4	8.91 ± 0.34	13.18 ± 1.65	20.22 ± 3.53	8.69 ± 1.25	27.597	<0.0001^a^
—	IL-2/IL-4	0.64 ± 0.14	41.82 ± 4.69	25.65 ± 5.57	0.71 ± 0.22	123.031	<0.0001^a^

a *p* < 0.0001.

The ratio of IL-2 and IL-4 is usually considered as a balance of locally vaginal homeostasis. For this reason, the ratio of IL-2/IL-4 in A431 cells exposed to *C. albicans* was calculated after treatment with or without BBR. Our results showed that the proportion of IL-2/IL-4 in A431 cells exposed to *C. albicans* significantly increased with the extension of exposure time (Table 1; *p* < 0.001), while it is worth noting that the increase in the ratio of IL-2/IL-4 was much smaller after treatment with BBR. At the same time point, the proportion of IL-2/IL-4 in A431 cells exposed to *C. albicans* after treatment with BBR was significantly lower than that in A431 cells only exposed to *C. albicans* without BBR treatment (Table 1; *p* < 0.001), indicating BBR could regulate the locally vaginal homeostasis by balancing the level of IL-2 and IL-4.

## Discussions

**FIGURE 7:**
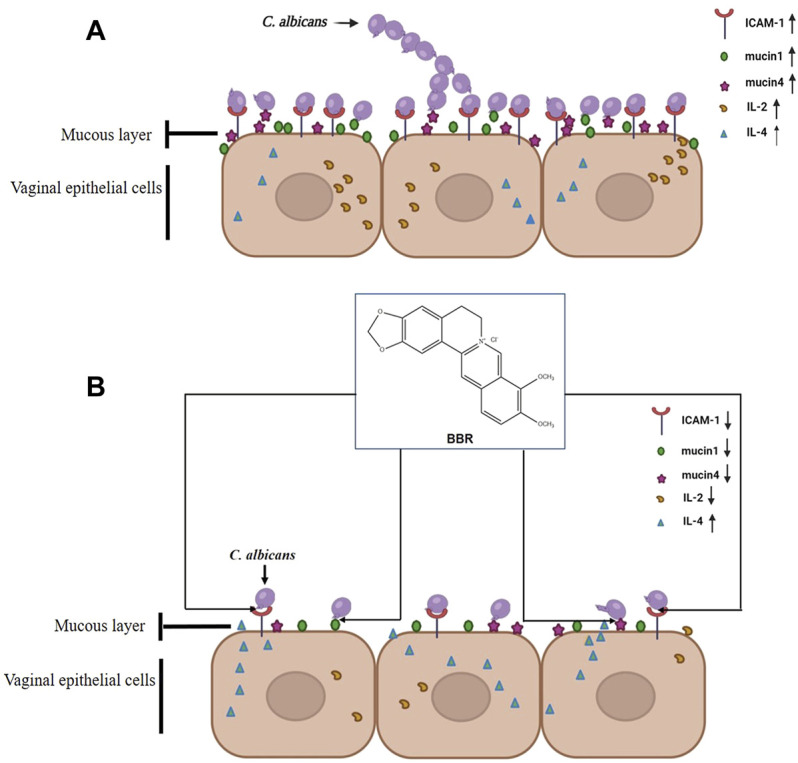
Possible mechanism of BBR blocking the adhesion of *C. albicans* to vaginal epithelial cells by inhibiting the expression of cell adhesion molecule ICAM-1, mucin1 and mucin4.

In this study, our data shows that BBR plays a key role in preventing the adhesion of *C. albicans* to vaginal epithelial cells. The expression of adhesion-associated molecules, such as ICAM-1, mucin1, and mucin4 significantly increased in vaginal epithelial cells when challenged with *C. albicans,* while they dramatically decreased after treatment of BBR. Consistently, the adhesion number of *C. albicans* to vaginal epithelial cells significantly decreased. The expression of pro-inflammatory cytokine IL-2 and anti-inflammatory cytokine IL-4 was regulated by this compound. A new immune homeostasis was rebuilt again. The inflammatory pathogenesis of vaginal epithelial cells was significantly improved. Thus, all of the data included in this paper imply that BBR could play an essential role in controlling infection of *C. albicans* during VVC pathogenesis through its effects on adhesion and regulation of immune barrier. Also, our study provides an alternatively potential strategy to develop new VVC therapy agents with relatively low toxicity from natural herbs (Figure 7).

Vulvovaginal candidiasis (VVC) is a mucosal immune inflammatory disease caused by *C. albicans* infection in the vagina due to pathogen-host interaction. Höfs and others found that some virulence factors of *C. albicans* such as candidalysin, Sap, and LP may play an important role in the development of VVC (Höfs et al., 2016). However, the effects of these virulence factors depend to a large extent on the interaction between pathogen and the host. It is required for *C. albicans* to establish infection that *C. albicans* adheres to vaginal epithelial cells. This adhesion is a complex interaction that involves a variety of signal molecules, including pathogen ligands, especially, pathogen-associated molecular pattern (PAMP), receptors, or adhesion molecules of host cells (Gilmore et al., 1988; Moyes et al., 2015). The adhesion-associated molecules such as ICAM-1, mucin-1, and mucin-4 are often highly expressed in vaginal epithelial cells following exposure to some pathogens, including *C. albicans* (Youn et al., 2008; Hinderfeld and Simoes-Barbosa 2020). ICAM-1 is an endothelial- and leukocyte-associated transmembrane protein whose main function is to stabilize the interaction between cells and promote the migration of leukocytes and endothelial cells (Lyck and Enzmann 2015; Bui et al., 2020). More recently, ICAM-1 has been characterized as a site for the epithelial adherance of fungi (Egusa et al., 2005; Mikamo et al., 2018). Because of these associations with immune responses, it has been considered as a pro-inflammatory factor that participates in inflammatory leukocyte recruitment and activation, and endothelial cell injury by signaling through cascades involving a number of kinases (Luo et al., 2020). ICAM-1 ligation enhances the adhesion of *C. albicans* to human vaginal epithelial cells (Mikamo et al., 2018). In this study, our data shows that ICAM-1 in vaginal epithelial cell line A431 increases during the exposure to *C. albicans*. Consistently, the adhesion number of *C. albicans* to cells also significantly increased. This suggests that the interaction between ICAM-1 on vaginal epithelial cell and its ligands from *C. albicans* could be a crucial step for the opportunistic infection caused by *C. albicans*. Thus, blocking this adhesion of *C. albicans* to vaginal epithelial cells may be a prospective strategy for the treatment of VVC. Of note, biofilm formation is recognized as an important virulence factor of *C. albicans*, which is responsible for chronic or recurrent infection, especially in catheter-related infection. More recently, the two groups had reported that BBR could inhibit the formation of *C. albicans* biofilms (Huang et al., 2020; Xie et al., 2020). Therefore, we did not repeat the experiment with BBR combating *C. albicans* biofilm in this study. In addition, the role of *C. albicans* biofilms in the pathogenesis of VVC is controversial, and Swidsinski A and others argued against the effects of *C. albicans* biofilms in VVC (Swidsinski et al., 2019). Taken together, the present study did not perform biofilm assay.

Mucins have been found to have important functions in defense against fungal infections, which exhibit unique gel properties that facilitate the mucus layer to adhere permanently to the epithelial cells, thereby protecting the epithelial cells from the invasion of fungi (Linford 1974; Zhao et al., 2020). Recently, mucins are considered as a biomarker of infectious vaginal diseases when they are excessively produced (Hinderfeld and Simoes-Barbosa 2020). As the classical members of the mucin family, mucin-1 and mucin-4 have been reported to have important biological functions on cell-cell and cell-extracellular matrix interactions, cell signal transduction, and cell carcinogenesis. Our previous results have shown that these two molecules are highly expressed in the vaginal mucosa of mice during VVC pathogenesis, and then significantly decreased after treatment with the extract of Pulsatilla chinensis (Bunge) Regel [Ranunculales] decoction (Zhao et al., 2020). Consistent with this result, our data showed that mucin-1 and mucin-4 were transiently highly expressed in vaginal epithelial cells in a time-dependent manner at the beginning of *C. albicans* adhesion; however, their expression was significantly reduced after BBR intervention. This reduction may be due to the fact that the compound BBR decreases ICAM-1 in the vaginal epithelial cells exposed to *C. albicans*, initiates a cascade reaction, and thereby activating a signal pathway.

BBR, a natural isoquinoline alkaloid with low toxicity, has been used for thousands of years to treat gastrointestinal diseases in China. However, in recent years, plenty of studies have shown that this compound has a wide spectrum of biochemical and pharmacological effects and it has been applied clinically in an attempt to combat some diseases such as diabetes, cancer, Alzheimer’s disease. Multiple mechanisms could be involved. For example, Liu and others confirm that BBR can prevent primary peritoneal adhesion and the adhesion reformation by directly inhibiting TIMP-1 in rats (Liu et al., 2020). However, Zhang and others find that this compound not only prevents post-surgery intestinal adhesion by down-regulating ICAM-1, but it also reduces inflammation by inhibiting the TAK1/JNK and TAK1/NF-κB signaling in rats (Zhang et al., 2014). Furthermore, studies also finds that it can protect the kidneys of diabetic nephropathy rats (Tang et al., 2016) and attenuate monocyte adhesion to endothelial cells induced by oxidized low-density lipoprotein by down-regulating ICAM-1 (Huang et al., 2013). In this study, we found that BBR has the minimal toxicity to vaginal epithelial cell line A431 at the concentration of 32 μg/ml and significantly inhibits the adhesion of *C. albicans* to these cells. Western blot further visualizes BBR significantly reduces the expression of ICAM-1in these cells, indicating that BBR could block the adhesion of *C. albicans* to vaginal epithelial cells mainly by modulating the expression ICAM-1, although this compound has a certain degree of antimicrobial effect. Besides, we also notice that this compound can down-regulate the expression level of mucins, suggesting that it could have the protection of vaginal epithelial cells from *C. albicans* invasion. This protective effect was also confirmed in the study of bacterial infections that BBR can inhibit bacterial adhesion and inflammation through decreasing mucin expression via the NF-κB signaling pathway, thereby protecting IPEC-J2 cells from enterotoxigenic *Escherichia coli* (ETEC) infection (Liu et al., 2019).

When infection occurs, lymphocytes and some immune-related cells such as epithelial cells often need to maintain a balance between type 1 and type 2 immunity to control the progress of the infection (Spellberg and Edwards 2001). It is well-known that IL-2 and IL-4, respectively, are the classical members of Type 1 and type 2 immunity. They play a crucial role in the regulation of local mucosal immunity. Therefore, correcting the imbalance of IL-2 and IL-4 caused by fungal infection may contribute to the restoration of local immune homeostasis. Baofukang suppository, a traditional Chinese medicine, has been shown to improve the clinical symptoms in VVC patients. *In vitro* experiments have observed that this medication can resume the *C. albicans*-triggered imbalanced ratio of IL-2/IL-4 cytokines to normal, resulting in the decrease of pro-inflammatory cytokine IL-2 and the increase of anti-inflammatory cytokine IL-4 (Li et al., 2016). Further, Lin and others confirmed that BBR down-regulates ratios of the relative IL-2/IL-4 cytokines expression fold in mouse primary splenocytes in the absence or presence of LPS in a preventive manner, suggesting that BBR may possess anti-inflammatory potential by shifting the Th1/Th2 balance toward Th2 polarization Lin and Lin, 2011. In this study, we observed that, following the exposure of *C. albicans* to vaginal epithelial cells, the expression of IL-2 is gradually increased, and IL-4 also increased. This increase could aim to counteract instinctively the effects of IL-2 at the very early stage of *C. albicans*. However, after treatment with BBR, IL-2 gradually decreased, while IL-4 gradually increased, indicating that BBR could play an important role in correcting cytokine imbalance during the pathogenesis of VVC.

## Conclusion

In this study, our data showed that BBR can decrease the adhesion of *C. albicans* and act as a potential therapeutic agent for VVC by inhibiting the expression of ICAM-1, mucin1, and mucin4, as well as alleviating inflammatory injury in vaginal epithelial cells by down-regulating the expression of IL-2 and by up-regulating the expression of IL-4.

## Data Availability

The raw data supporting the conclusions of this article will be made available by the authors, without undue reservation.
